# Notes and News

**Published:** 1899-02-11

**Authors:** 


### Notes and News.

(Collected and Communicated.)

HOSPITAL MEETING.

St. Mary's Children's Hospital, Plaistow.—Annual Court, Thursday, February 16th, at 5 p.m., at the Hospital.

The Cavendish lecture for 1899 will be delivered by Professor Osler, F.R.S., of the Johns Hopkins University, Baltimore, before the West London Medico-Chirurgical Society.

The Queen will shortly open the new children's wing of the Royal Isle of Wight County Hospital. The addition to the hospital will serve as a Jubilee Memorial, and on the occasion of the opening Princess Beatrice will unveil a bust of the Queen.

The Bromley Cottage Hospital has received the munificent gift of £4,000 from Mr. J. Bennett, of Keston, to endow a ward in memory of his son, to be named after him. The ward, which contains eight cots, was originally benefited by Mr. Bennett's generosity, who gave £525 towards its erection.

The danger of oysters as typhoid-carriers has not yet disappeared, though the scare created some two years ago has abated. A paper by Professors Herdman and Boyer before the Royal Society, the result of careful investigation, places the danger with the retailer rather than the oyster. As is well known, sea water is inimical to the typhoid bacillus, and when oysters come direct from the markets and the sea they are generally healthy, but the reverse is frequently the case with those bought from shops, and in those that have been kept or "fattened" in sewage polluted water.

Opinions conflict at Southampton over the matter of a proposed expenditure by the Guardians on a new infirmary. It appears that the new institution is intended to house 300 sick paupers; and the best defence urged by those who appear to favour the scheme for so large a building was that the Local Government Board had insisted that the accommodation should be for this number. It is a matter of speculation in some quarters where the 300 sick are coming from, seeing that 200 inmates is regarded as a large number at the present time. Southampton is also about to build an isolation hospital, at a large expenditure.

At the annual meeting of the Peterborough Infirmary it was proposed to increase the medical staff by the appointment of an assistant surgeon, and it was decided that a special meeting should be called at which the necessary alteration of the rules might be made.

The National Health Society has just issued a new edition of its "Facts Concerning Vaccination." This useful little penny pamphlet, which is intended for distribution among heads of families, has been altered to suit the arrangements under the new Act, and can be purchased in quantities at a cheap rate.

At an influential meeting of the medical committee held recently, Dr. Green in the chair, it was unanimously resolved that the open-air treatment of tuberculosis be tried at the Brompton Hospital, and the subject is now under consideration by the Committee of Management, together with that of the establishment of a sanatorium in the country.

Dr. Goodhart is making a further appeal on behalf of Dr. Reichardt, who incurred expenses amounting to £700 in defending an action brought by the Commissioners in Lunacy to convict him of misdemeanour for receiving a patient who was suffering from a mental ailment, but whom neither Drs. Kingsford, Savage, nor Goodhart would certify. The Commissioners contended that Dr. Reichardt should not have received such a patient uncertified. Dr. Goodhart points out that Dr. Reichardt was defending the right of everyone to do their best to treat an individual patient according to his needs. The friends of patients who, although mentally ill, are not so definitely deranged as to be fit for a certificate of insanity, are in a sad position if they cannot procure the assistance of the constant care of a medical man Dr. Goodhart, at 25, Portland Place, and Dr. Savage, of 3, Henrietta Street, W., will receive contributions to assist Dr. Reichardt.

A year ago last January Lady Elgin laid the foundation-stone of the Lady Dufferin Victoria Hospital, Bengal's Jubilee memorial. In January of this year she declared the institution open, this being the last public ceremonial in which she took part before her departure from India. The old Dufferin Hospital, which had proved too small, was sold, the proceeds being devoted to procuring the site of the new one. Many influential native personages have contributed handsome sums towards the new building, the main portion of which is now completed. At the opening ceremony it was stated that three additional wards were required, namely, for maternity, ophthalmic, and contagious cases; and it was announced that the Viceroy had received promises of 21,000 rupees towards these on the day of the opening. In the main building the large central ward is paved with slabs of marble, the verandahs with hard concrete. All the openings are screened with shielding venetian blinds. The roof is arranged as a promenade or airing space, and is of easy access from the wards. The operating-rooms are provided with glazed tiled walls, and all the sanitary arrangements have been carefully planned. In the entrance stands a bust of Lady Dufferin.

